# Durable Intracranial Control Beyond Five Years in EGFR Wild-Type Non-Small Cell Lung Cancer with Sequential Brain Metastases Managed with Multimodal Therapy: A Case Report

**DOI:** 10.3390/reports9030220

**Published:** 2026-07-10

**Authors:** Mihai-Teodor Georgescu, Andrada Maria Bărbuț

**Affiliations:** 1 Oncology 2 Discipline, Department 8, Faculty of Medicine, “Carol Davila” University of Medicine and Pharmacy, 050474 Bucharest, Romania; 2 Radiotherapy 2, “Prof. Dr. Alexandru Trestioreanu” Oncology Institute, 022328 Bucharest, Romania; andradamariabarbut@yahoo.com

**Keywords:** non-small cell lung cancer, brain metastases, stereotactic radiosurgery, whole-brain radiotherapy, neurosurgical resection, multimodal treatment, EGFR wild-type, long-term survival, case report

## Abstract

**Background and Clinical Significance**: Brain metastases in non-small cell lung cancer (NSCLC) carry a poor prognosis, particularly in patients lacking targetable driver mutations or significant programmed death-ligand 1 (PD-L1) expression. Durable intracranial control exceeding five years is uncommon in this population and the factors that determine exceptional therapeutic response remain incompletely understood; **Case Presentation**: We report a 59-year-old male with pathological stage pT3N1 solid-type pulmonary adenocarcinoma (EGFR wild-type, ALK wild-type, PD-L1 <1%) who developed two sequential brain metastases following right upper lobectomy and adjuvant pembrolizumab plus pemetrexed-carboplatin. The first lesion was treated with single-fraction stereotactic radiosurgery (SRS, 10 Gy); a second metastasis identified 18 months later was managed with focal radiotherapy (8 Gy, single fraction) followed by whole-brain radiotherapy (24 Gy in 12 fractions). Local progression of the second metastasis in 2024 prompted successful surgical resection via right occipital craniotomy. Over a follow-up exceeding five years, the patient achieved sustained intracranial disease control, preserved neurological function, and maintained quality of life. Notably, no clinically apparent neurocognitive deterioration was documented on routine clinical follow-up, despite whole-brain irradiation without hippocampal sparing; formal neuropsychological testing was not performed; **Conclusions**: This case demonstrates that durable intracranial control may be achievable through carefully sequenced multimodal therapy—including stereotactic radiosurgery, whole-brain radiotherapy, and neurosurgical resection—even in biologically unfavorable NSCLC. The absence of clinically apparent neurocognitive deterioration on routine follow-up after WBRT raises hypothesis-generating questions regarding interindividual variability in radiation tolerance; this observation must be interpreted in the absence of formal neuropsychological testing and prospective hippocampal dosimetry. A multidisciplinary, individualized approach integrating radiotherapy, systemic therapy, and neurosurgery remains essential in this setting.

## 1. Introduction and Clinical Significance

Brain metastases arise in 25–35% of patients with systemic malignancies and are up to ten-times more frequent than primary brain tumors. Among all solid tumors, lung carcinoma is the predominant source, accounting for approximately 30–60% of all intracranial metastatic lesions [1,2]. The prognosis for affected patients has historically been poor, with median survival under six months in unselected populations, though modern multimodal strategies—encompassing stereotactic radiosurgery (SRS), surgery, immune checkpoint inhibitors (ICIs), and targeted agents—have begun to extend survival in defined subgroups [1,3].

The molecular profile is a central determinant of both metastatic risk and therapeutic opportunity in NSCLC. Epidermal growth factor receptor (EGFR) mutations are associated with an elevated propensity for CNS dissemination but confer sensitivity to CNS-penetrant tyrosine kinase inhibitors [4]. Conversely, patients who are EGFR wild-type, ALK wild-type, and have low PD-L1 expression occupy a particularly challenging therapeutic space: they lack targeted agents with established intracranial activity and frequently derive limited benefit from immunotherapy alone. Long-term intracranial disease control in this subgroup is uncommon and, when it occurs, poses important hypothesis-generating questions about the determinants of individual treatment response [5,6,7]. 

We report a 59-year-old male with pT3N1, G3 solid-type pulmonary adenocarcinoma—EGFR wild-type, PD-L1 <1%—who developed two sequential brain metastases and remained disease-free in the central nervous system for more than five years, managed through stereotactic radiosurgery, whole-brain radiotherapy, and neurosurgical resection. The case is clinically relevant because long-term intracranial control is uncommon in this molecular subgroup, and the reasons for this favorable course cannot be determined from a single case.

## 2. Case Presentation

### 2.1. Initial Presentation and Primary Tumor Management

A 59-year-old male former smoker (35 pack-years; smoking cessation approximately 10 years prior to diagnosis), with known stage 2 arterial hypertension, presented to an outside facility in February 2020 with progressive dyspnea. Thoracic CT demonstrated a spiculated mass in the right upper lobe extending to the horizontal fissure, mediastinal lymphadenopathy with oncologic characteristics (17 mm in the Barety space; subcentimetric nodes in the aortopulmonary window), obstruction of the right upper lobar bronchus, and right-sided thoracic osteophytosis, without pleural effusion (Figure 1).

At presentation, the patient had an ECOG performance status of 1. He underwent right upper lobectomy with mediastinal lymphadenectomy and partial apical pleurectomy. Postoperative histopathology confirmed solid-type pulmonary adenocarcinoma, G3 (TTF-1+, CK7+, Chromogranin A−, Synaptophysin−, CD56−), staged pT3pN1 with 4/4 lymph nodes involved. Molecular analysis showed absence of EGFR and ALK mutations; PD-L1 expression was <1%. Broader next-generation sequencing was not available at the time of diagnosis, and retrospective NGS analysis of archived surgical specimens was not feasible due to institutional biobanking constraints. Adjuvant systemic therapy consisted of six cycles of pembrolizumab, pemetrexed, and carboplatin.

### 2.2. First Brain Metastasis: Diagnosis and Radiotherapy

In May 2020—three months after surgery—contrast-enhanced brain MRI revealed a mixed-structure lesion with a cystic component and a peripherally enhancing solid nodule at the left temporo-parietal junction (32 × 26 × 5 mm axially; 34 mm craniocaudally), surrounded by extensive digitiform edema causing mass effect on the left lateral ventricle (Figure 2A,B). The findings were radiologically consistent with a metastatic lesion. Concurrent thoracoabdominal CT showed tumor recurrence at the apico-posterior pleura with chest wall invasion and contiguous osteolysis of T2–T3 vertebral bodies, the T2 pedicle, the right T3 transverse process, and the posterior third rib.

Between 5 June and 12 June 2020, the patient received external beam radiotherapy. For the cerebral lesion, stereotactic treatment was delivered using a linear accelerator with image-guided capabilities (IGRT). Gross tumor volume (GTV) was defined on contrast-enhanced MRI fused with the planning CT and corresponded to the enhancing lesion; planning target volume (PTV) was generated with minimal margin expansion per institutional stereotactic protocol. A single fraction of 10 Gy was prescribed to the isodose line encompassing the PTV. Organs at risk—including the brainstem, optic apparatus, and uninvolved brain parenchyma—were contoured and dose constraints applied in accordance with contemporary SRS guidelines. The 10 Gy single-fraction dose, while below contemporary ablative SRS targets (typically 18–24 Gy for lesions <2 cm), was selected given the lesion measured 32 mm axially with extensive surrounding edema; the treating team prioritized safety and edema control over dose escalation.

For the site of chest wall and vertebral recurrence (T2–T3), conformal external beam photon radiotherapy was delivered to a total dose of 20 Gy in four fractions (5 Gy per fraction, one fraction per day). Treatment was well tolerated with marked improvement in thoracic pain; cerebral edema resolved under concomitant corticosteroid and analgesic therapy.

### 2.3. Second Brain Metastasis: Focal Radiotherapy Followed by Whole-Brain Radiotherapy

In 21 September 2021—18 months after the first intracranial event—restaging CT identified a right occipital nodular lesion with ring enhancement (0.8 × 0.7 cm), with perilesional edema and mass effect on the posterior horn of the right lateral ventricle, consistent with a second metastatic lesion. Over the preceding 4–6 weeks, the patient had developed progressive occipital headache and visual disturbances, including blurred vision and intermittent right homonymous hemifield complaints, consistent with the anatomical location of the lesion. Symptoms were of moderate severity, interfering with daily activities.

Between 22 November and 14 December 2021, the patient received a two-phase radiotherapy course. Phase 1: a single fraction of 8 Gy was delivered to the right occipital metastasis using conformal photon radiotherapy. GTV corresponded to the ring-enhancing lesion on planning MRI; dose was prescribed to the encompassing isodose line with standard stereotactic margin. Phase 2: therapeutic whole-brain radiotherapy (WBRT) was administered to a total dose of 24 Gy in 12 fractions (2 Gy per fraction, five fractions per week) using conventional opposed lateral fields. Hippocampal-sparing techniques were not employed. The 8 Gy single-fraction dose for the right occipital metastasis reflected its proximity to critical posterior fossa structures; dose escalation was not pursued given these anatomical constraints and the decision to proceed immediately to WBRT. Prospective dose-volume histogram (DVH) data for hippocampal structures were not recorded at the time of planning. Based on published dosimetry for conventional opposed lateral WBRT at 24 Gy, mean hippocampal dose would be expected to approach or exceed the 7.3 Gy constraint recommended in hippocampal-avoidance protocols (NRG CC001); the clinical significance of this in the absence of formal neuropsychological testing cannot be determined. Treatment was well tolerated; neurological symptoms resolved fully.

It bears emphasis that the WBRT delivered in this case was therapeutic—administered in response to a confirmed second metastasis—rather than prophylactic in the strict sense. Whether WBRT contributed to eradicating subclinical micrometastatic disease at other sites remains a plausible, though unproven, hypothesis. At follow-up assessments, the patient reported no memory complaints, and clinical and functional assessment—including maintained ECOG performance status of 1—revealed no evidence of treatment-related neurocognitive deterioration. No seizures were documented. Formal neuropsychological testing was not performed; this represents a limitation of the present report.

### 2.4. Progression and Neurosurgical Resection

A follow-up brain MRI in March 2024 demonstrated dimensional progression of the right occipital lesion (2.2 × 1.3 cm compared with 1.6 cm on prior examination; Figure 3), prompting multidisciplinary review. In April 2024, the right occipital metastasis was resected via right occipital craniotomy in accordance with standard operative protocol. The postoperative course was uneventful with no new neurological deficits. Histopathological analysis of the resected specimen confirmed metastatic adenocarcinoma consistent with the known lung primary (TTF-1+, CK7+). No features suggesting histological transformation or novel characteristics that might independently explain the prolonged clinical course were identified. Comparative molecular analysis of paired specimens (2020 primary vs. 2024 metastasis) was not performed, as tissue was not prospectively preserved for research; this is acknowledged as a limitation.

### 2.5. Outcome and Current Status

The most recent brain MRI—obtained in March 2025, more than five years after the initial diagnosis—demonstrated slight regression of the right occipital resection cavity compared with the post-surgical examination (Figure 4). No new intracranial lesions were identified. At the time of reporting, the patient maintains a markedly improved clinical condition, ECOG performance status of 1, preserved neurological function, and acceptable quality of life.

## 3. Discussion

This case raises two points worth discussing. First, a patient without standard targetable molecular alterations—EGFR wild-type, ALK wild-type, PD-L1 <1%—remained free of intracranial progression for more than five years. This outcome is longer than expected for this clinical profile. Second, whole-brain radiotherapy was delivered without hippocampal protection, yet no neurocognitive decline was apparent on routine clinical assessment, though this was not formally tested.

A few published series describe long-term survivors after multimodal treatment for NSCLC brain metastases, but most include patients with targetable alterations, higher PD-L1 expression, or limited intracranial disease. This patient had none of these favorable features. Whether the favorable outcome reflects tumor biology, a contribution from immunotherapy, the treatment sequence, or chance cannot be determined.

To provide comparative survival context: Sperduto et al. (2020) reported in a large pooled prospective dataset that estimated median survival for patients with EGFR/ALK wild-type NSCLC brain metastases and a diagnosis-specific Graded Prognostic Assessment (GPA) score of 1.5–2.0—consistent with this patient’s profile (pT3pN1, G3, PD-L1 <1%, ECOG 1, single brain metastasis at first event)—is approximately 9–11 months from diagnosis of brain metastases [8]. Estimated 5-year survival in this GPA tier is approximately 3–8%. Survival exceeding five years in this prognostic category substantially exceeds population-level expectations, supporting the characterization of this outcome as exceptional while acknowledging that comprehensive molecular profiling was unavailable.

The molecular profile of this patient placed him at multiple intersecting disadvantages. EGFR wild-type status precludes the use of CNS-penetrant tyrosine kinase inhibitors (e.g., osimertinib), which have transformed intracranial disease control in EGFR-mutated NSCLC [4]. PD-L1 <1% expression predicted limited immunotherapy efficacy, consistent with the modest objective response rates observed in this biomarker subgroup with pembrolizumab-based regimens [9]. Simultaneously, the patient harbored multiple established risk factors for CNS dissemination: pT3pN1 staging, G3 histology, solid adenocarcinoma subtype, tumor size >3 cm, and nodal involvement—each independently associated with elevated brain metastasis risk in resected NSCLC [10,11,12]. That sustained intracranial control was ultimately achieved in this context is a meaningful observation, suggesting that molecular risk stratification alone does not preclude durable intracranial disease control in selected patients.

An important and under-discussed factor in this case is the potential contribution of adjuvant immunotherapy. The patient received six cycles of pembrolizumab plus pemetrexed-carboplatin. Although PD-L1 <1% is associated with limited pembrolizumab monotherapy benefit, the KEYNOTE-189 combination regimen demonstrated overall survival improvement even in the PD-L1 <1% stratum (hazard ratio approximately 0.59 vs. chemotherapy alone) [9]. Emerging data suggest that immune checkpoint inhibitors may prime durable systemic and potentially intracranial immune responses persisting beyond the treatment period—the concept of immune memory—and that focal radiation may enhance this effect through immunogenic tumor cell death and the abscopal phenomenon [13]. It is therefore plausible, though unconfirmable from this single case, that pembrolizumab-mediated immune priming contributed to prolonged intracranial disease stability, either directly or by sensitizing residual tumor to subsequent local therapies. This possibility underscores that exceptional outcomes in PD-L1-low patients should not reflexively exclude an immune contribution.

The sequential nature of metastatic events in this case—a first brain metastasis at three months and a second at 18 months postoperatively—is consistent with epidemiological data showing that CNS recurrence risk increases progressively with higher pathological stage and tumor grade, persisting well beyond the perioperative window [14]. The absence of EGFR mutations, while negating access to targeted CNS therapy, did not confer a uniformly poor prognosis in this individual— a finding that resonates with emerging evidence that current biomarkers capture only part of the biological variance underlying individual treatment response in EGFR wild-type disease [5].

From a radiotherapy standpoint, single-fraction SRS (10 Gy for the first metastasis; 8 Gy for the second) was selected for both initial metastatic events, in accordance with the guideline-supported approach for limited intracranial disease [15,16]. The relatively conservative single-fraction doses reflected lesion size, anatomical location, and institutional practice at the time, prioritizing treatment safety and edema control over ablative dosing—a reasonable approach given the large, edema-associated first lesion and the proximity of the second to critical posterior fossa structures. When the second metastasis developed in the context of prior focal CNS irradiation, the decision to follow focal treatment with therapeutic WBRT reflected the cumulative intracranial disease burden and the risk of subclinical micrometastatic dissemination. Whether WBRT contributed to preventing further CNS events—a plausible but unverified hypothesis—cannot be established from a single case. Clinically, no new intracranial lesions were documented during the 18 months following WBRT, and the period of greatest neurological stability coincided with its completion. The subsequent local progression of the right occipital metastasis in 2024 may reflect suboptimal initial local control from the 8 Gy single fraction—a possibility that cannot be excluded and is acknowledged as a treatment-planning limitation. In contemporary practice, dose escalation to 15–18 Gy with careful posterior fossa OAR constraint optimization would represent a preferable approach for similarly located lesions.

The absence of clinically apparent neurocognitive deterioration on routine follow-up warrants specific attention. Radiation-induced hippocampal injury has been mechanistically linked to cognitive decline in patients receiving WBRT, and hippocampal-sparing techniques are increasingly incorporated into contemporary practice to mitigate this risk [17]. The fact that this patient did not develop clinician-documented neurocognitive complaints and maintained stable ECOG performance status throughout and after WBRT is consistent with, though not equivalent to, preserved neurocognitive function. In the absence of serial neuropsychological testing, we note the absence of clinically evident impairment as assessed by the treating team, while explicitly acknowledging that subtle cognitive changes cannot be excluded. This observation does not argue against the routine use of hippocampal-sparing WBRT where feasible; rather, it underscores that population-level risk estimates carry substantial individual-level variance. Serial neuropsychological assessments using validated instruments such as the Hopkins Verbal Learning Test-Revised (HVLT-R) and the Controlled Oral Word Association test (COWA) would be required to detect domain-specific deficits; these were not performed at baseline or follow-up in this patient. Furthermore, in the absence of prospective hippocampal DVH data, the actual dose delivered to hippocampal structures cannot be quantified; based on published dosimetry for conventional WBRT, mean hippocampal dose would be expected to approach or exceed the 7.3 Gy constraint associated with memory decline in hippocampal-avoidance trials, and the absence of clinical neurotoxicity in this context should not be interpreted as evidence of hippocampal radio-tolerance.

Surgical resection of the progressing right occipital lesion in 2024—following prior SRS and WBRT—exemplifies the role of neurosurgery as a salvage modality in selected patients with radiologically confirmed progression, good performance status, and limited intracranial disease [18]. The successful postoperative course and subsequent imaging-confirmed regression illustrate that surgical options remain viable even late in a complex treatment trajectory and should be routinely considered at multidisciplinary tumor board review.

Taken together, this case shows that some patients in a poor-prognosis subgroup achieve unexpectedly long intracranial disease control. The reasons are not clear from the available data. The case supports the value of individualized reassessment at each step rather than a fixed treatment sequence. Prospective collection of molecular and dosimetric data in future long-term survivors would help identify factors associated with this type of outcome.

Several directions are relevant to future cases of this type. SRS dose and fractionation continue to be refined, and higher single-fraction doses may be feasible for posterior fossa lesions with modern planning tools. The potential role of immunotherapy in intracranial disease control, including in PD-L1-low patients, warrants prospective study. Cerebrospinal fluid liquid biopsy is an emerging tool for monitoring intracranial disease and may be useful in future cases of sequential brain metastases [19,20,21]. 

This report has several limitations. First, as a single case, no generalizable conclusions about treatment efficacy can be drawn, and the favorable outcome may reflect chance or unidentified biological factors. Second, molecular characterization was limited to EGFR, ALK, and PD-L1; broader next-generation sequencing—including KRAS, STK11, KEAP1, MET exon 14, RET, ROS1, and NTRK—was not available at diagnosis, and retrospective analysis of archived tissue was not feasible due to institutional biobanking constraints. Third, comparative molecular profiling of the 2020 primary tumor and the 2024 metastatic specimen was not performed. Fourth, formal neuropsychological testing (e.g., HVLT-R, COWA) was not performed at any time point; all statements about cognitive function are based on clinical observation only. Fifth, prospective dose-volume histogram data for hippocampal structures were not recorded during radiotherapy planning.

## 4. Conclusions

We report a case of durable intracranial disease control exceeding five years in a patient with biologically unfavorable NSCLC—EGFR wild-type, PD-L1 <1%, pT3N1 G3 adenocarcinoma—managed through a sequenced strategy of SRS, therapeutic WBRT, and neurosurgical resection. Several observations from this case are hypothesis-generating and merit discussion in the broader clinical context.

First, high-risk molecular and pathological characteristics do not preclude durable intracranial disease control in selected patients. The absence of driver mutations that would have provided targeted CNS treatment options did not prevent sustained disease control in this case, raising questions about what alternative biological factors—intrinsic tumor characteristics, immune microenvironment, host factors—may have contributed to the observed outcome.

Second, the lack of clinically evident neurocognitive deterioration during routine follow-up after conventional WBRT without hippocampal sparing is a noteworthy descriptive observation but should be interpreted cautiously in the absence of formal serial neuropsychological assessment, as subtle cognitive changes cannot be excluded. This observation does not diminish the population-level evidence supporting hippocampal-avoidance strategies.

Third, the multidisciplinary, longitudinal decision-making evident in this case—with treatment re-evaluated and adapted at each disease event over five years—illustrates the practical importance of ongoing specialist collaboration in the management of CNS metastatic disease.

Continued research into biomarkers of intracranial treatment response, refined radiotherapy techniques, and integrated systemic-local strategies remains essential for improving outcomes in this complex and biologically diverse patient population.

## Figures and Tables

**Figure 1:**
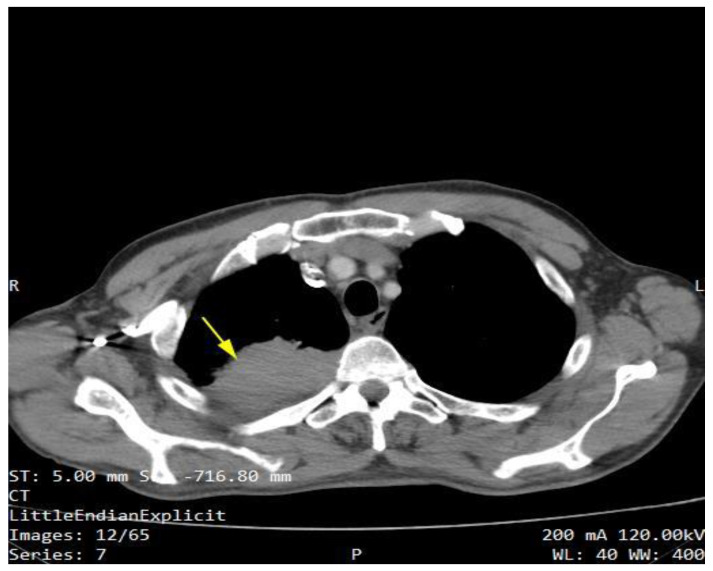
Thoracic CT demonstrating the primary tumoral mass (yellow arrow) in the right upper lobe with spiculated margins extending to the horizontal fissure, with associated mediastinal lymphadenopathy.

**Figure 2:**
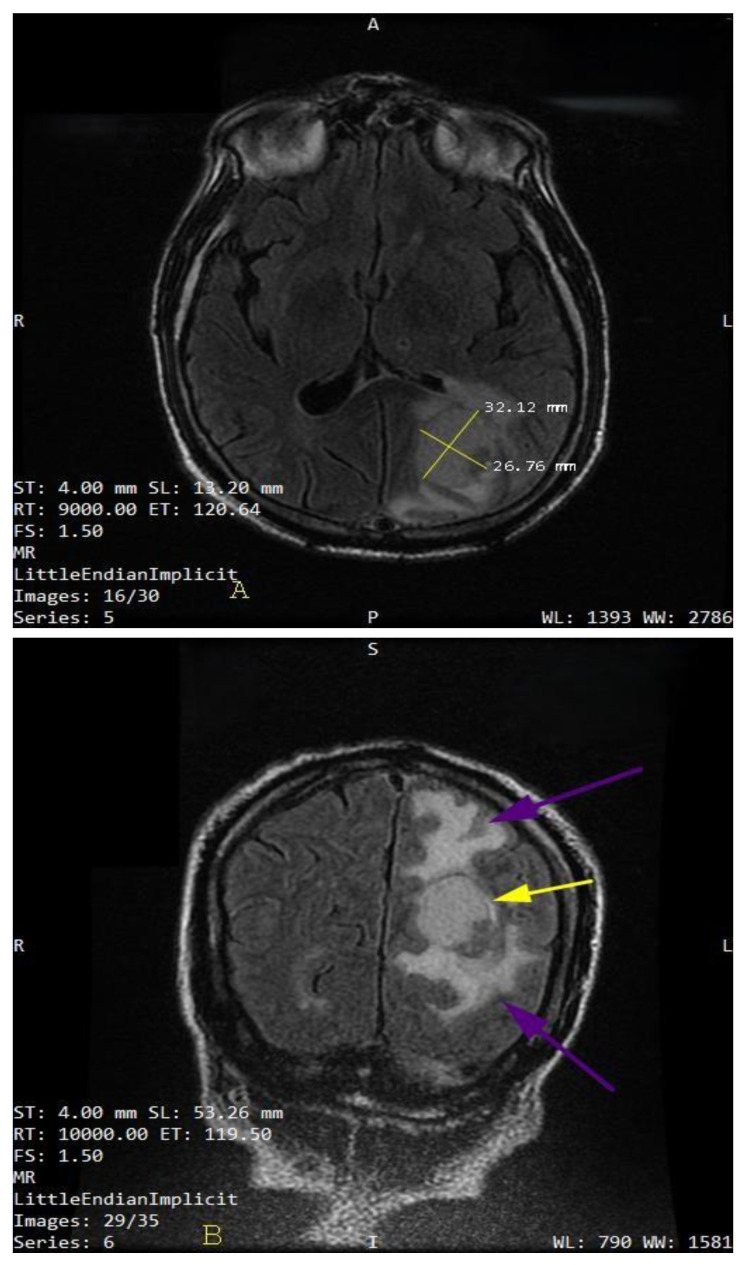
MRI of the first brain metastasis at the left temporo-parietal junction. (**A**) Axial view. (**B**) Coronal view showing the metastatic lesion (yellow arrow) surrounded by diffuse digitiform edema (purple arrows).

**Figure 3:**
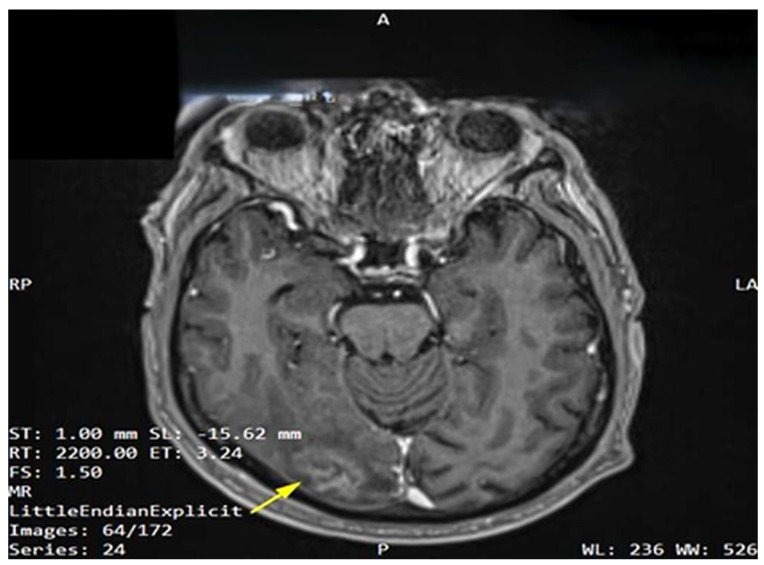
Brain MRI (March 2024) demonstrating dimensional progression of the right occipital metastatic lesion (yellow arrow) compared with prior imaging, prompting surgical review.

**Figure 4:**
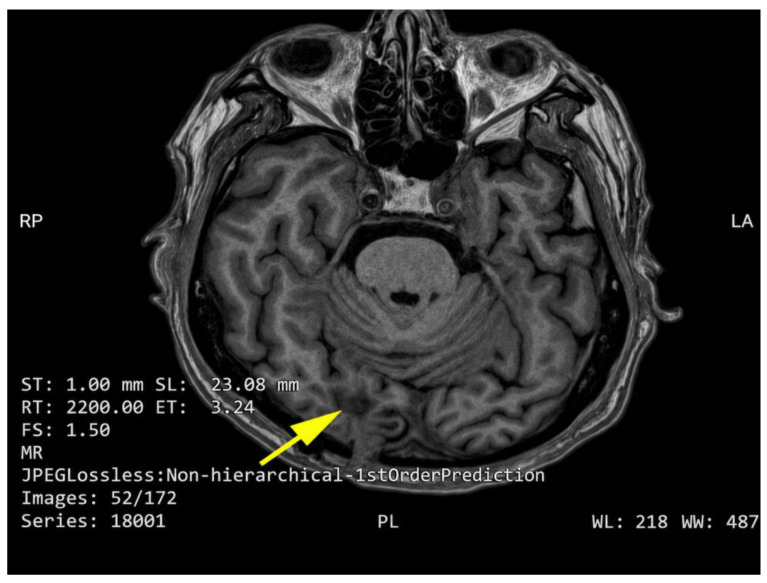
Follow-up brain MRI (March 2025), axial view. Slight regression of the right occipital lesion (yellow arrow) compared with prior imaging, consistent with ongoing local disease control.

## Data Availability

The data supporting the findings of this case report are available from the corresponding author upon reasonable request.
